# Supplementary material for: Asymmetric nucleosome PARylation at DNA breaks mediates directional nucleosome sliding by ALC1
Source: Nat Commun. 2024 Feb 2;15:1000. doi: 10.1038/s41467-024-45237-8 (PMC10837151; doi:10.1038/s41467-024-45237-8)
Supplement: Supplementary file 1 — Supplementary Information [file 41467_2024_45237_MOESM1_ESM.pdf]

Asymmetric nucleosome PARylation at DNA breaks mediates directional nucleosome sliding by ALC1

Luka Bacic, Guillaume Gaullier, Jugal Mohapatra, Guanzhong Mao, Klaus Brackmann, Mikhail Panfilov, Glen Liszczak, Anton Sabantsev, and Sebastian Deindl

Supplementary Information Content:

Supplementary Figure 1. Related to Figure 1. Unprocessed original gel images
Supplementary Figure 2. Related to Figure 2. Overview of cryo-EM image analysis
Supplementary Figure 3. Related to Figure 2. Occupancy of the features in our cryoEM reconstruction.
Supplementary Figure 4. Related to Figure 3. SmFRET example traces and remodeling initiation time
Supplementary Figure 5. RP-HPLC and ESI-MS characterization of ADP-ribosylated histones generated in this study.
Supplementary Table 1. Sequences of oligonucleotides used in the study.

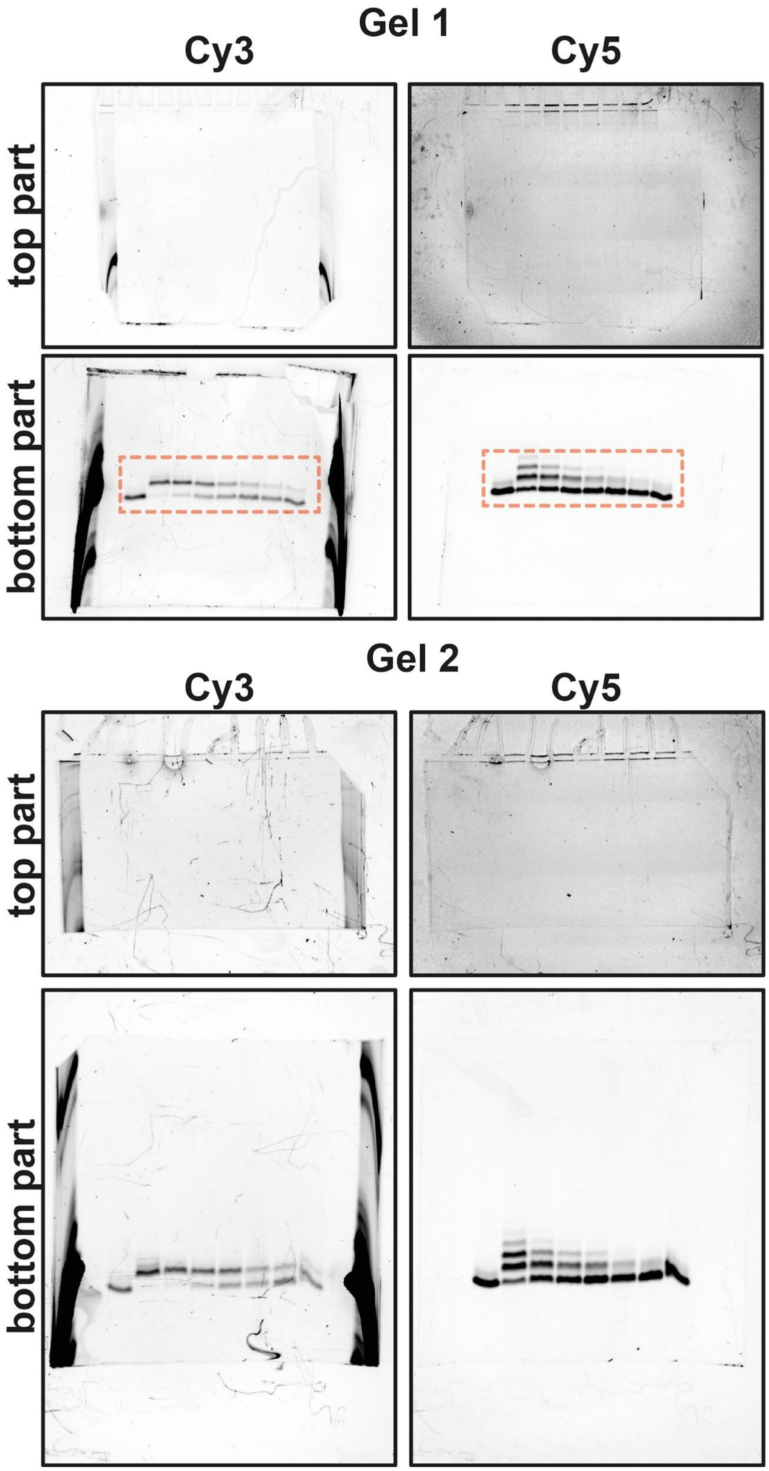

Supplementary Figure 1. Related to Figure 1. Unprocessed original gel images.
SDS-PAGE gels imaged by Cy3 and Cy5 fluorescence detection, resolving the histones after PARylation by PARP1/HPF1 at different NAD^+ concentrations, as specified in Figure 1b. Cy3-labeled H2A histone was overloaded in the outer lanes as a reference to visually follow the migration. Two independent repeats are shown. The area highlighted with a semi-transparent dashed red rectangle is shown in Figure 1b.

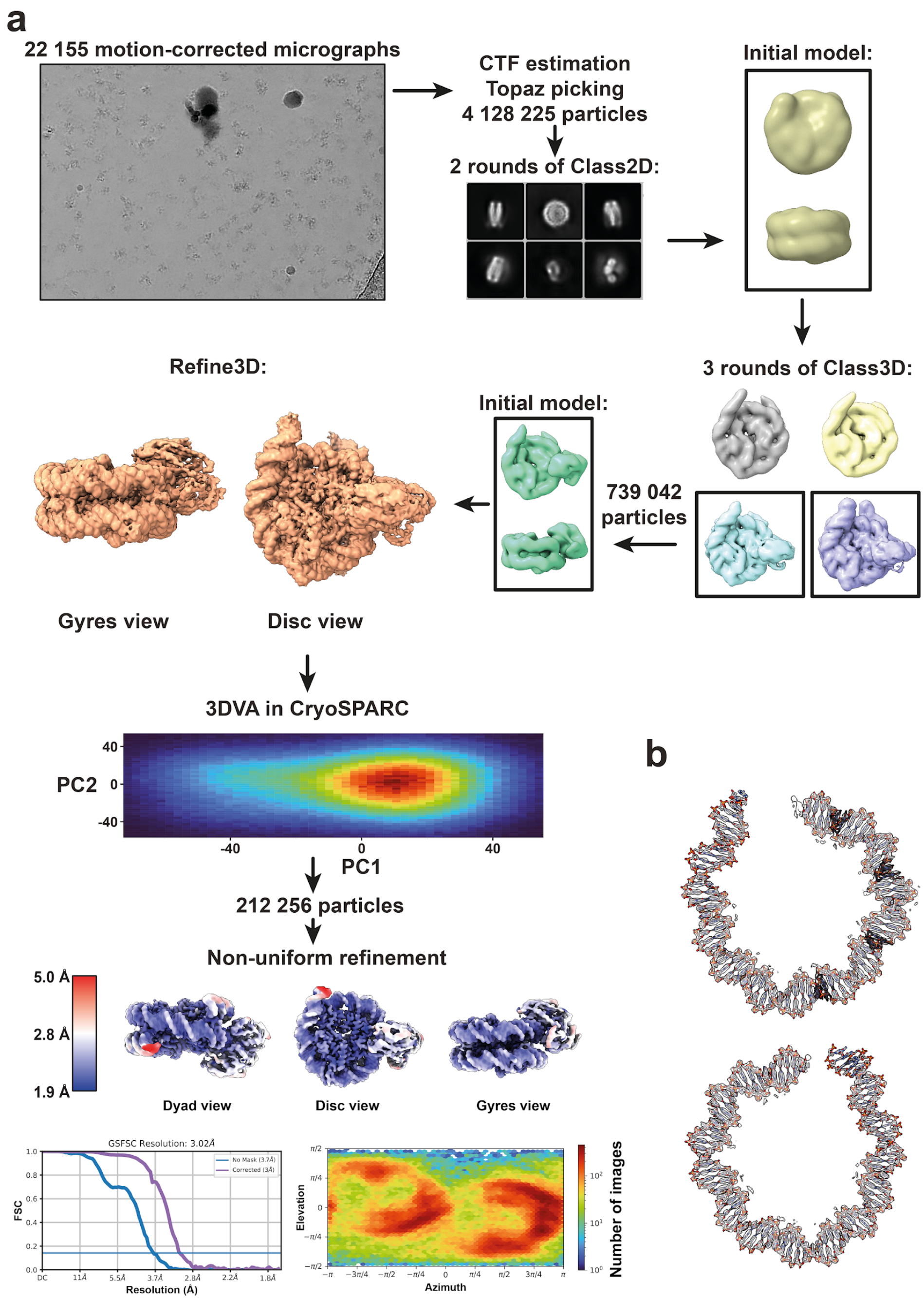

Supplementary Figure 2. Related to Figure 2. Overview of cryo-EM image analysis.
a, Flow chart of the cryo-EM image processing strategy. **b**, Model-to-map fit of the DNA, with atomic model shown as sticks and map shown as translucent surface (at a contour level of 0.4). For clarity, the two halves of the nucleosomal DNA are shown separately. Top: strong side of the 601 sequence, with the four TA steps colored in black. Bottom: weak side of the 601 sequence.

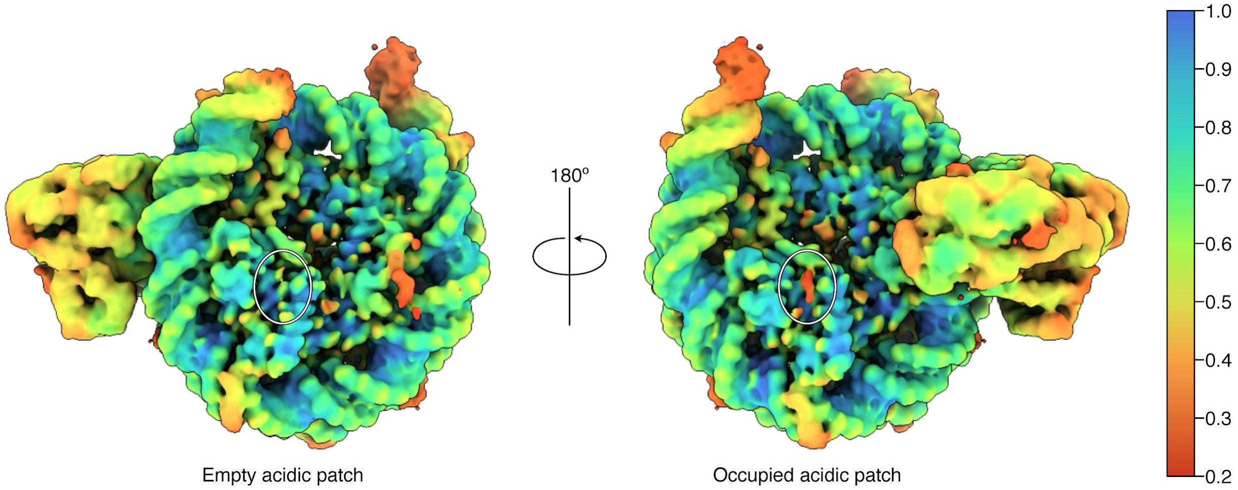

Supplementary Figure 3. Related to Figure 2. Occupancy of the features in our cryoEM reconstruction. The map is displayed at a contour level of 0.055 and colored by occupancy as quantified by OccuPy. Both sides of the nucleosome are shown and the acidic patch is circled. The acidic patch is visibly bound by the Arg anchor of ALC1 (albeit at low occupancy, consistent with a dynamic interaction of the ALC1 linker with the acidic patch) only on the strong (entry) side of the nucleosome.

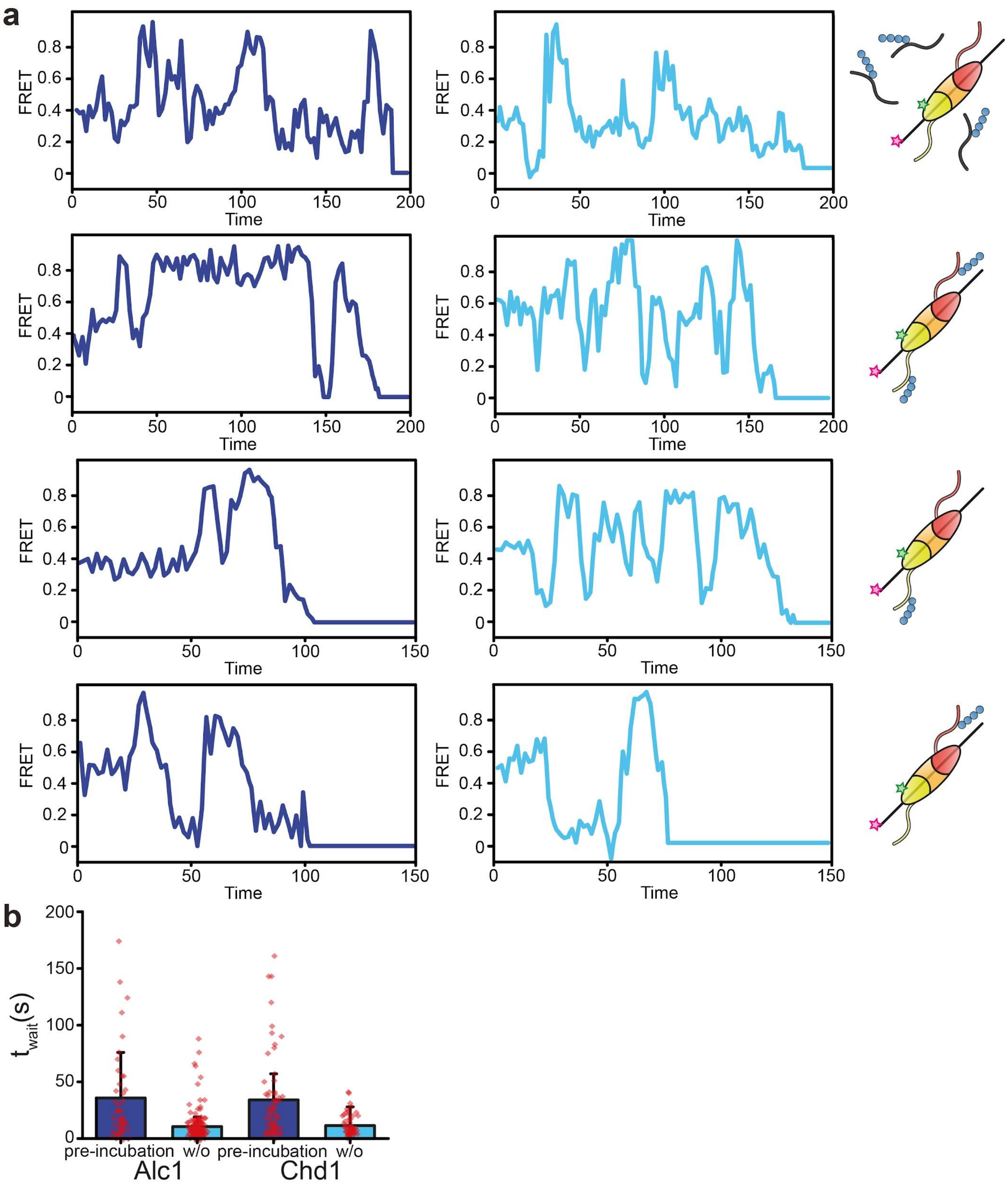

Supplementary Figure 4. Related to Figure 3. SmFRET example traces and remodeling initiation time.

a, Example FRET traces from individual nucleosomes that are initially remodeled by ALC1 towards the short DNA linker (dark blue) or away from it (light blue). **b**, Remodeling initiation time for ALC1 (light blue) and Chd1 (dark blue) with and without preincubation with remodeler in the absence of ATP (Mean±SEM, n = 51, 160, 62 and 58 traces). Individual data points are presented as red diamonds. ALC1 experiments were carried out with 1 μM ALC1 and 1 mM

ATP. Chd1 experiments were carried out with 480 nM Chd1 and 10 μM ATP. Source data are provided as a Source Data file.

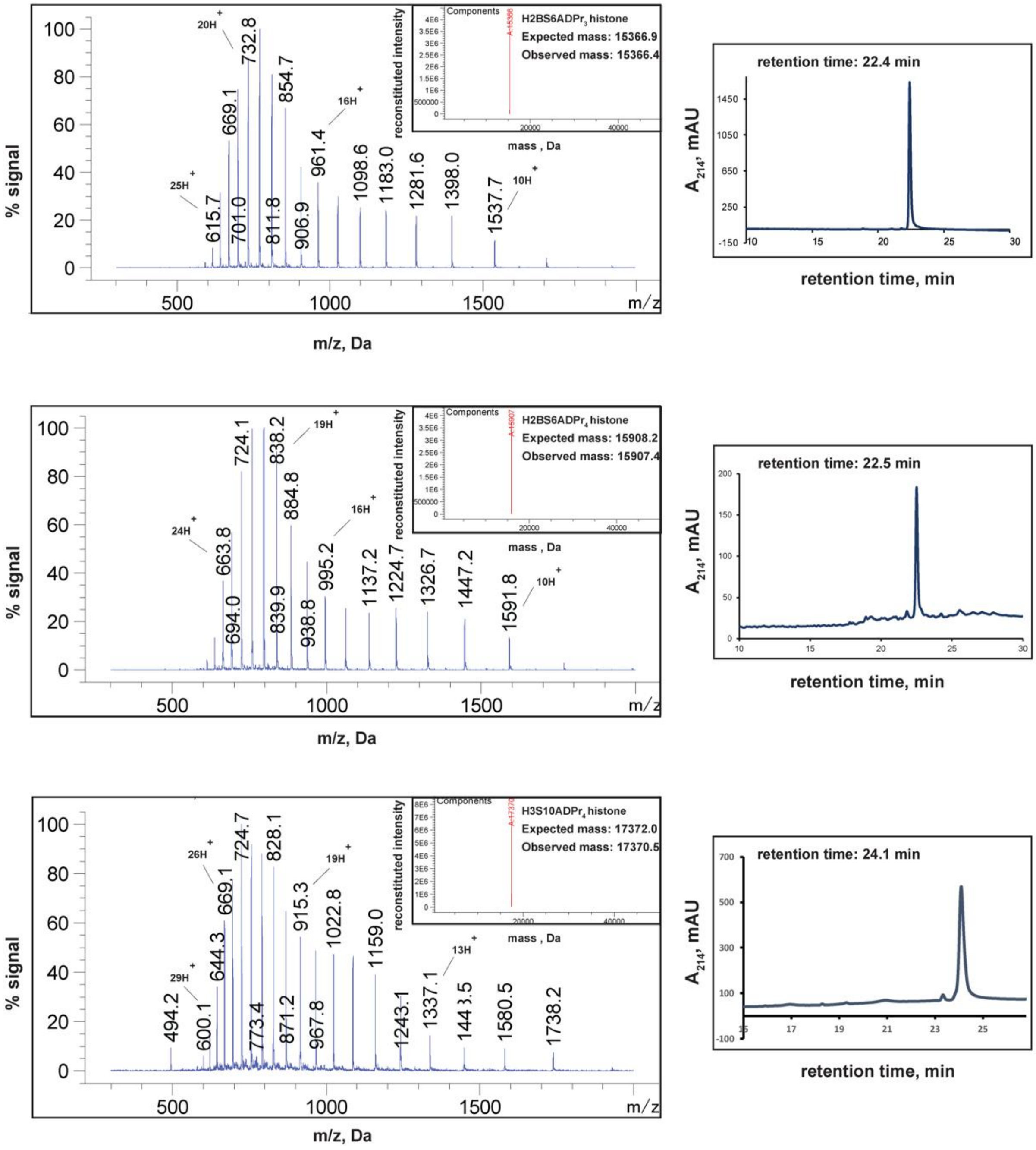

Supplementary Figure 5. RP-HPLC and ESI-MS characterization of ADP-ribosylated histones generated in this study.

The RP-HPLC gradient is 0-80% Solvent B from 2-32 min for the H2B histones and 0-70 % Solvent B from 5-25 min for the H3 histone

Name	Sequence
Cryo-EM_F	TCTAGGTGACCATCAGAATCCCGGTGCC
Cryo-EM_R	AATCGATGTATATATCTGACACGTGCCTGGAGACTAGGG
PARylation_F	GCCCTGGAGAATCCCGGTCTGCA
PARylation_R	GGTACCCGTAGATCCTCTAGAGTGGGAGCTC
+12_core_F_Cy5	/5Cy5/TACGCGGCCGCCCTGGAGAATCCCGG
+12_core_R_BsaI	TTTTTTGGTCTCAGACTAGGGAGTAATCCCCTTGGC
+12_stem_F_BsaI	AAAAAAGGTCTCTAGTCTCCAGGCACGTGTCA
+12_stem_R_Bio	/5BiotinTEG/GGTACCCGTAGATCCTCTAGAGTGGGAGCTCG

Supplementary Table 1. Sequences of oligonucleotides used in the study.
